# Parent Race and Communication During Elective Pediatric Surgery Consultations

**DOI:** 10.1001/jamanetworkopen.2025.42758

**Published:** 2025-11-11

**Authors:** Chenery Lowe, Mary Catherine Beach, Somnath Saha, Anne R. Links, Emily F. Boss

**Affiliations:** 1 Center for Biomedical Ethics, Stanford University, Stanford, California; 2 Department of Otolaryngology-Head and Neck Surgery, Johns Hopkins University, Baltimore, Maryland; 3 Division of General Internal Medicine, Department of Medicine, Johns Hopkins University, Baltimore, Maryland

## Abstract

**Importance:**

Child and adolescent members of marginalized racial groups have poorer surgical outcomes compared with White children and adolescents. Clinical communication may contribute to these disparities.

**Objective:**

To explore racial differences in parent-clinician communication during initial consultations for elective surgical procedures in children and adolescents.

**Design, Setting, and Participants:**

This cohort study investigated communication during consultations between parent-clinician dyads: surgical clinicians and parents of children and adolescents ages 2 to 17 years referred for initial evaluation for tonsillectomy or adenoidectomy, hernia repair, and circumcision between 2016 and 2023 at academically affiliated outpatient clinics in the Baltimore, Maryland, region.

**Main Outcomes and Measures:**

Visits were audio-recorded and coded using the Roter Interaction Analysis System, a quantitative communication coding system developed to analyze medical interactions. Outcomes included visit-level measures (parent vs clinician verbal dominance and patient-centeredness ratio), clinician communication (facilitation and activation statements to encourage parent participation, emotional statements, social chitchat, and normalized positive affect *Z* scores), and parent communication (emotional statements, social chitchat, and normalized positive affect *Z* scores). Generalized estimating equations were used to test associations of parent race with visit communication, accounting for nesting of visits within clinicians and adjusting for parent sociodemographic variables.

**Results:**

A total of 153 parents (63 Black [41.2%] and 90 White [58.8%]; mean [SD] age, 35.1 [6.7] years; 135 female [88.2%]) and 22 clinicians (2 Asian [9.1%], 1 Hispanic or Latino [4.5%], 13 White [59.1%], 3 another race or ethnicity [13.6%], and 3 multiple races or ethnicities [13.6%]; 12 female [54.5%]) were included. In unadjusted models, visits with Black parents had higher clinician verbal dominance (0.4 more clinician statements [95% CI, 0.1-0.8 statements] per parent statement) but no difference in overall patient-centeredness scores compared with visits with White parents. However, visits with Black parents had 2.3 fewer clinician chitchat statements (95% CI, 3.3 to 1.3 statements), 26.6 fewer total parent statements (95% CI, 46.0 to 7.2 statements), 2.1 fewer parent emotional statements (95% CI, 3.6 to 0.7 statements), and 1.7 fewer parent chitchat statements (95% CI, 2.8 to 0.55 statements) per visit and lower parent positive affect (*Z* score = −0.6; 95% CI, −1.0 to −0.2) compared with visits with White parents. After adjustment for parent sociodemographic variables, clinician verbal dominance remained significantly higher, with clinicians making 0.4 more statements (95% CI, 0.1-0.7 statements) per parent statement.

**Conclusions and Relevance:**

In this study, parent race was associated with differences in clinician, parent, and interactive visit communication, although some differences appeared to be mediated by sociodemographic factors and most were due to differences in parent rather than clinician communication. These findings suggest that application of patient-centered communication and engagement strategies may help bridge social distance in pediatric surgical care.

## Key Points

**Question:**

How does parent-clinician communication differ by parent race during initial pediatric surgery consultations?

**Findings:**

In this cohort study among 153 parents and 22 surgical clinicians, consultations with Black parents had higher clinician verbal dominance, less parent emotional expression, and less social chitchat for parents and clinicians compared with visits with White parents.

**Meaning:**

These findings suggest opportunities to mitigate racial differences in communication and bridge social distance in pediatric surgical care, including through improved clinician elicitation of patient and family concerns and intentional attempts to build rapport.

## Teaser

This cohort study investigates the association of parent race with parent-clinician communication during pediatric elective surgery consultations.

## Introduction

In a 2013 meta-analysis,^1^ Black children in the US had higher rates of postoperative mortality and morbidity and longer hospital stays compared with White children even after adjusting for comorbidities. Multiple factors contribute to this disparity, likely including patient-parent communication. In other settings including adult primary care, clinician bias is associated with less patient-centered communication with patients from marginalized racial and ethnic backgrounds.^2,3,4^ In pediatric surgery, this raises concerns about whether parents from marginalized groups are sufficiently able to discuss their concerns about risks, benefits, and options when making surgical decisions. There is limited evidence about racial differences in communication during pediatric surgical consultations.

Our objective was to explore racial differences in communication during initial consultations for 3 common referral indications: tonsillectomy or adenoidectomy, hernia repair, and circumcision. We hypothesized that visits with Black parents would have higher clinician verbal dominance and lower patient-centeredness compared with visits with White parents. We explored associations of race with specific aspects of clinician communication (facilitation and activation statements, social chitchat, emotional statements, and global affect) and parent communication (social chitchat, emotional statements, and global affect).

## Methods

The Johns Hopkins School of Medicine Institutional Review Board (IRB) approved all procedures and was the IRB of record for this cohort study. Participants completed written informed consent before engaging in study activities. We followed the Strengthening the Reporting of Observational Studies in Epidemiology (STROBE) reporting guideline.

### Participants and Data Collection

Data were collected at 3 outpatient otolaryngology clinics, 1 urology clinic, and 1 general surgery outpatient clinic at Johns Hopkins Medicine from 2016 to 2023 as part of a study evaluating communication, shared decision-making, and bias in pediatric elective surgery consultations. Clinicians were eligible if they were an attending physician or advanced practice clinician who regularly evaluated children and adolescents for tonsillectomy or sleep-disordered breathing, hernia repair, or circumcision. Statements from supporting clinicians (eg, residents and other clinical staff) were included in recordings and coding, but demographics for these clinicians were not available. Parents were eligible if their child underwent initial evaluation for sleep-disordered breathing or tonsillectomy, hernia, or circumcision. Parents were identified via electronic health records and contacted before their visit. After providing informed consent, participants completed demographic questionnaires. Consultations were audio-recorded.

### Parent, Child, and Clinician Sociodemographic Variables

The parent sociodemographic questionnaire included self-reported questions about parent age, parent gender, parent race, parent ethnicity, annual household income, parent education, insurance type, child procedure, child gender, child age, child race, and child ethnicity. Clinicians reported their gender, race, ethnicity, and years of experience. Race response options included American Indian or Alaska Native, Asian, Black or African American, Native Hawaiian or Other Pacific Islander, White, and other. Ethnicity options included no, not of Hispanic, Latino, or Spanish origin; yes, Mexican, Mexican American, or Chicano; yes, Cuban; and yes, another Hispanic, Latino, or Spanish origin. Respondents could select multiple options for race and ethnicity. Individuals selecting *other* in response to race or *yes, another Hispanic, Latino, or Spanish origin* in response to ethnicity could specify in a free-response box. For this analysis, parents were considered to be non-Hispanic or Latino White (hereafter, *White*) if their selected race was *White* and ethnicity was *no, not of Hispanic, Latino, or Spanish origin* and Black if they selected their race as *Black or African American*. For clinicians, race responses were not used if the clinician selected *Hispanic, Latino, or Spanish origin*. Parents were excluded from this analysis if they selected *White* and *Hispanic, Latino, or Spanish origin*.

### Communication Coding

A single coder with more than 10 years of experience coded visit recordings using the Roter Interaction Analysis System (RIAS), a widely used quantitative communication coding system developed for medical interactions.^5^ The coder did not view participant surveys but was not blinded to demographic information that could be determined from listening to the recording. Coder drift was assessed by recoding and calculating intracoder reliability for a random sample of visits, but reliability data were not available at the time of this interim analysis. RIAS coding categorizes each unit of clinician or patient expression that conveys a complete thought into 1 of 37 mutually exclusive and exhaustive codes reflecting biomedically and socioemotionally focused statements.^5^ Codes were combined to produce categories described subsequently, with definitions and examples in Table 1.

**Table 1.** Parent and Clinician Communication Behaviors and Examples

Category	RIAS code	Example and definition
Visit variables		
Verbal dominance	NA	Ratio of all clinician to all parent statements
Patient-centeredness ratio	NA	Ratio including the sum of: Clinician psychosocial questions, psychosocial information, emotionally responsive statements, and facilitation and activation statements Parent psychosocial questions, psychosocial information, emotionally responsive statements, and medical questions Divided by: Clinician clinical questions and medical and therapeutic information-giving Parent medical and therapeutic information-giving
Clinician behaviors		
Facilitation and activation statements	Ask for patient opinion Ask for patient understanding Paraphrase Back-channel (cues of interest)	“So do you have a preference in what way we go?” “Does that make sense?” “…sounds like the sleep is impacting her during the day.” “Right.”
Emotionally responsive statements	Concerns Reassurance and optimism Empathy and legitimation Partnership Self-disclosure	“Sorry about that.” “Generally, removing the tonsils is pretty effective.” “I know it’s really tough.” “Let me know how I can help you.” “In my 3-year-old, I would do a partial [tonsillectomy].”
Social chitchat	Nonmedical social chitchat	“Yeah, [city name] has changed over time.”
Positive global affect	Ratings on a scale of 1-6 of the following attributes: Interest and attentiveness Friendliness and warmth Responsiveness and engagement Sympathetic and empathic qualities Respectfulness	Mean ratings were normalized as study-specific *Z* scores
Parent behaviors		
Emotional expression	Concerns Reassurance and optimism Asks for reassurance Empathy and legitimation	“It was really painful.” “We are going on a better path now.” “Do you think it’s serious?” “Many people feel the same way.”
Social chitchat	Nonmedical social chitchat	“I graduated from there.”
Positive global affect	Ratings on a scale of 1-6 of the following attributes: Interest and attentiveness Friendliness and warmth Responsiveness and engagement Sympathetic and empathic qualities Respectfulness	Mean ratings were normalized as study-specific *Z* scores

Abbreviations: NA, not applicable; RIAS, Roter Interaction Analysis System.

#### Visit-Level Measures

Coded statements were combined to calculate verbal dominance, the ratio of all clinician statements to all patient statements during the visit. Codes were also combined to calculate the patient-centeredness ratio, which reflects the balance between socioemotional and biomedical exchange, with higher values indicating a stronger socioemotional focus.

#### Clinician Communication Behaviors

Codes were combined to capture clinician facilitation and activation statements, emotionally responsive statements, and social chitchat. These categories are subsets of socioemotionally focused communication. When more than 1 clinician spoke during a visit, coding distinguished between primary and supporting clinicians, typically an advanced practice clinician or trainee. To ensure that analyses captured the patient’s entire experience of the consultation, RIAS outcome variables reflect communication with primary and supporting clinicians.

#### Parent Communication Behaviors

RIAS codes were combined to create categories reflecting parent emotional expression and social chitchat. Coding did not distinguish between speakers when more than 1 adult caregiver was present during a visit.

#### Affect Ratings

After coding each visit, the coder rated clinician and parent affect during the visit using a Likert scale ranging from 1 to 6. Positive clinician and parent affect were each determined by calculating the mean of global ratings across 5 dimensions: interest and attentiveness, friendliness and warmth, responsiveness and engagement, sympathetic and empathetic qualities, and respectfulness. Due to the potential for coder drift, these ratings were normalized as *Z* scores based on mean affect ratings from each study data–collection period.

### Statistical Analysis

Analyses included parents who identified as Black or White. Because parents of other races and ethnicities represented a small proportion of the study population (29 of 182 individuals [15.9%]), we lacked sufficient sample size to conduct comparative analyses by parent race. Bivariate analyses assessed the unadjusted association of parent race with outcomes listed in Table 1. Differences in sociodemographic characteristics by parent race were assessed using analysis of variance for continuous variables (parent, child, and clinician age) and χ^2^ tests for categorical variables. We used generalized estimating equations with an exchangeable correlation structure to evaluate the association of parent race with each communication behavior, accounting for clustering within clinicians. Because race can be associated with outcomes directly and indirectly (eg, through association with other forms of privilege or disadvantage), we assessed unadjusted models and models that adjusted for parent age, gender, insurance, and education (trichotomized as high school or less, some college or 2-year degree, or 4-year degree or any postgraduate education). Because annual household income and health insurance type were associated, only insurance type was included as a model covariate because it was hypothesized to be more closely associated with health care access. Both measures were associated with parent race. Missing data were considered to be missing completely at random. Reasons for missing data were generally unrelated to participant or visit characteristics and include inability to code the visit due to poor quality audio recording or need for additional processing, technical issues with the recording, multiple children and adolescents being evaluated during the visit (13 of 166 visits [7.8%]), and noncompletion of survey measures (Table 2). Analyses were conducted using R statistical software version 4.3.1 (R Project for Statistical Computing).^6^ The level of significance was *P* < .05; *P* values and tests were 2-sided.

**Table 2.** Visit Characteristics

Characteristic	Visits, No. (%)			*P* value
	Black or African American parent (n = 63)	White parent (n = 90)	Total (n = 153)	
**Parent characteristics**				
Age, mean (SD), y	33.6 (6.8)	36.1 (6.5)	35.1 (6.7)	.02
Gender				
Female	59 (93.7)	76 (84.4)	135 (88.2)	.08
Male	4 (6.3)	14 (15.6)	18 (11.8)	
Annual household income, $				
No. with data	59	87	146	NA
<20 000	23 (39.0)	3 (3.4)	26 (17.8)	<.001
20 000-49 999	22 (37.3)	9 (10.3)	31 (21.2)	
50 000-79 999	6 (10.2)	15 (17.2)	21 (14.4)	
80 000-100 000	3 (5.1)	14 (16.1)	17 (11.6)	
>100 000	5 (8.5)	46 (52.9)	51 (34.9)	
Not reported, No.	4	3	7	
Highest level of education				
No. with data	61	90	151	NA
Did not graduate high school	4 (6.6)	0	4 (2.6)	<.001
High school or GED	18 (29.5)	4 (4.4)	22 (14.6)	
Some college or 2-y degree	21 (34.4)	27 (30.0)	48 (31.8)	
Graduated college or 4-y degree	13 (21.3)	23 (25.6)	36 (23.8)	
Master’s, professional, or doctoral degree	5 (8.2)	36 (40.0)	41 (27.2)	
Not reported, No.	2	0	2	
Health insurance type				
No. with data	61	88	149	NA
Employer-based, private, or commercial	14 (23.0)	68 (77.3)	82 (55.0)	<.001
State funded or medical assistance	47 (77.0)	20 (22.7)	67 (45.0)	
Not reported, No.	2	2	4	
Procedure type				
Tonsillectomy or adenoidectomy	55 (87.3)	78 (86.7)	133 (86.9)	.19
Circumcision	6 (9.5)	4 (4.4)	10 (6.5)	
Hernia	2 (3.2)	8 (8.9)	10 (6.5)	
Child gender				
No. with data	62	90	152	NA
Female	32 (51.6)	27 (30.0)	59 (38.8)	.007
Male	30 (48.4)	63 (70.0)	93 (61.2)	
Not reported, No.	1	0	1	
Child age, mean (SD), y	5.91 (3.1)	5.22 (3.6)	5.50 (3.4)	.22
Child race and ethnicity				
Asian	0	2 (2.2)	2 (1.3)	<.001
Black and African American	61 (96.8)	0	61 (39.9)	
White	0	76 (84.4)	76 (49.7)	
Multiple races or ethnicities	2 (3.2)	12 (13.3)	14 (9.2)	
**Visit-level characteristics of clinicians**				
Gender				
Female	35 (55.6)	47 (52.2)	82 (53.6)	.68
Male	28 (44.4)	43 (47.8)	71 (46.4)	
Race and ethnicity				
Asian	2 (3.2)	2 (2.2)	4 (2.6)	.07
Hispanic or Latino	2 (3.2)	1 (1.1)	3 (2.0)	
White	44 (69.8)	58 (64.4)	102 (66.7)	
Other^a^	9 (14.3)	9 (10.0)	18 (11.8)	
Multiple races or ethnicities, White	2 (3.2)	18 (20.0)	20 (13.1)	
Multiple races or ethnicities, Black/African American	3 (4.8)	2 (2.2)	5 (3.3)	
Multiple races or ethnicities, not specified	1 (1.6)	0	1 (0.7)	
Experience, y				
0-5	20 (31.7)	26 (28.9)	46 (30.1)	.32
6-10	11 (17.5)	20 (22.2)	31 (20.3)	
11-15	24 (38.1)	20 (22.2)	44 (28.8)	
16-20	1 (1.6)	7 (7.8)	8 (5.2)	
>20	7 (11.1)	17 (18.9)	24 (15.7)	

Abbreviations: GED, General Educational Development test; NA, not applicable.

^a^ Includes Asian American and Japanese American.

## Results

### Participant Characteristics

Of 182 parent-patient dyads enrolled in the study, 153 parents (84.0%) were Black or White. To facilitate statistical comparisons, visits with these 153 parents (63 Black [41.2%] and 90 White [58.8%]; mean [SD] age, 35.1 [6.7] years; 135 female [88.2%]) were included in analysis. The mean (SD) parent age was 33.6 (6.8) years for Black parents and 36.1 (6.5) years for White parents. In both race groups, most parents were female (59 female Black parents [93.7%] and 76 female White parents [84.4%]). Additional demographics are shown in Table 2. Compared with Black parents, White parents were older, had a higher annual family income, and had more education. White parents were more likely to have employer-based, private, or commercial health insurance rather than state-funded or medical assistance insurance and had a higher proportion of male compared with female children and adolescents being evaluated for surgery.

A total of 22 clinicians were included (12 female [54.5%]; 2 Asian [9.1%], 1 Hispanic or Latino [4.5%], 13 White [59.1%], 3 another race or ethnicity [13.6%], and 3 multiple races or ethnicities [13.6%] that included Black or African American for 1 participant and White for another participant). Because data were collected over multiple years, clinician age and years of experience varied. Table 2 shows characteristics of clinicians involved in the 153 consultations. Clinicians conducted study visits with a mean (SD) of 7.0 (7.9) enrolled families each (median [range], 3 [1-24] visits per clinician). A supporting clinician, such as a medical resident or nurse, participated in 99 visits (64.7%). Typically, the supporting clinician would see the family unsupervised before the attending physician joined the visit.

### Visit-Level Communication

There was no difference in mean (SD) total visit length by parent race (White parents: 18.0 [6.9] minutes; Black parents: 17.2 [6.7] minutes). Clinician verbal dominance was lower during visits with White parents compared with Black parents, indicating that clinician statements represented a larger share of the dialogue compared with parent statements (Table 3). This association was found in the unadjusted model (model 1), with clinicians making 0.4 more statements (95% CI, 0.1-0.8 statements) per parent statement in visits with Black parents compared with White parents. This association was also found after adjusting for parent age, gender, and insurance type (model 2: β = 0.5; 95% CI, 0.1-0.8) and after additionally adjusting for parent education (model 3: β = 0.4; 95% CI, 0.1-0.7). Models excluding dialogue between the supporting clinician and the parents found similar unadjusted and adjusted associations between parent race and verbal dominance (eTable in Supplement 1). Given that differences in verbal dominance may reflect clinicians talking more, parents talking less, or both, we explored the association of parent race with total clinician and parent statements separately. In the unadjusted model (model 1; Table 3), Black parents made 26.6 fewer total statements (95% CI, 46.0-7.2 statements) compared with White parents. While models that adjusted for parent demographics (models 2 and 3) found that visits with Black parents had fewer parent statements and more clinician statements, none of these individual differences were statistically significant. There were no differences by parent race for patient centeredness (Table 3).

**Table 3.** Association of Parent Race With Communication During Consultations

Outcome	Parent statements, mean (SD)		Coefficient (95% CI) compared with White		
	Black (n = 57)^a^	White (n = 85)^a^	Model 1^b^	Model 2^c^	Model 3^d^
Overall					
Verbal dominance^e^	1.8 (0.8)	1.4 (0.4)	0.4 (1.0 to 0.8)	0.5 (0.1 to 0.8)	0.4 (0.1 to 0.7)
Patient centeredness^f^	0.4 (0.1)	0.4 (0.1)	0.0 (0.0 to 0.0)	0 (0.0 to 0.0)	0.0 (0.0 to 0.0)
Clinician behavior					
Total statements	220.7 (98.6)	204.7 (84.2)	16.3 (−17.3 to 49.9)	27.8 (−13.2 to 68.8)	31.9 (−14.9 to 78.7)
Facilitation and activation	36.9 (27.1)	37.4 (19.9)	−0.2 (−5.5 to 5.1)	2.6 (−4.9 to 10.1)	3.8 (−4.8 to 12.4)
Emotional statements	11.7 (9.8)	9.8 (7.6)	1.8 (−1.7 to 5.3)	2.6 (−0.6 to 5.8)	3.1 (−0.6 to 6.8)
Chitchat	3.9 (3.6)	6.0 (5.3)	−2.3 (−3.3 to −1.3)	−1.3 (−2.8 to 0.3)	−0.9 (−2.4 to 0.6)
Positive affect (standardized)	−0.2 (0.9)	−0.1 (1.0)	−0.1 (−0.4 to 0.2)	−0.2 (−0.5 to 0.1)	−0.2 (−0.5 to 0.1)
Parent behavior					
Total statements	140.6 (79.7)	162.5 (82.3)	−26.6 (−46.0 to −7.2)	−17.3 (−46.4 to 11.8)	−8.5 (−39.6 to 22.6)
Emotional statements	6.3 (6.2)	7.9 (6.0)	−2.1 (−3.6 to −0.7)	−1.2 (−3.2 to 0.8)	−0.6 (−2.7 to 1.5)
Chitchat	2.6 (3.1)	4.3 (4.1)	−1.7 (−2.8 to −0.5)	−1.4 (−2.6 to −0.3)	−0.9 (−2.2 to 0.4)
Positive affect (standardized)	−0.3 (1.1)	0.1 (0.9)	−0.6 (−1.0 to −0.2)	−0.6 (−1.1 to −0.1)	−0.5 (−1.0 to 0.1)

^a^ Population numbers represent parents with complete responses for all sociodemographic variables included in statistical models.

^b^ Unadjusted.

^c^ Adjusted for demographics excluding education.

^d^ Adjusted for demographics including education.

^e^ Ratio of clinician to parent statements.

^f^ Ratio of socioemotionally focused statements to task-focused statements.

### Clinician Communication

Clinician social chitchat was lower during visits with Black compared with White parents, with clinicians making 2.3 fewer chitchat statements (95% CI, 3.3-1.3 statements) per consultation with Black parents in the unadjusted model (model 1; Table 3). After adjusting for parent characteristics (models 2 and 3), there was no statistically significant difference in clinician chitchat statements by parent race. There were no differences in clinician facilitation or activation statements or emotionally responsive statements (Table 3).

### Parent Communication

Black parents made 1.7 fewer chitchat statements (95% CI, 2.8 to 0.55 statements) than White parents in the unadjusted model (model 1; Table 3) and in a model that adjusted for parent age, gender, and insurance (model 2: β = −1.4; 95% CI, −2.6 to −0.3). However, racial difference was no longer significant after adjusting for parent education (model 3: β = −0.9; 95% CI, −2.2 to 0.4). Black parents made 2.1 fewer emotional statements (95% CI, 3.6 to 0.7 statements) than White parents in the unadjusted model (model 1; Table 3). However, after adjusting for parent demographics, we found no association between parent race and emotional statements (models 2 and 3).

### Clinician and Parent Affect

Internal consistency across positive affect categories was adequate for parent (standardized Cronbach α = 0.8; 95% CI, 0.81 to 0.89) and clinician (standardized Cronbach α = 0.82; 95% CI, 0.75 to 0.87) ratings. White parents had higher positive affect ratings compared with Black parents in the unadjusted model (model 1; *Z* score = −0.6; 95% CI, −1.0 to −0.2) and in the model that adjusted for parent age, gender, and insurance type (model 2; *Z* score = −0.6; 95% CI, −1.1 to −0.1). There was no association after adjustments for parent education (model 3). There were no racial differences in clinician positive affect in adjusted or unadjusted models (Table 3).

## Discussion

While parent race was not associated with many communication behaviors assessed in this cohort study, visits with Black parents had higher clinician verbal dominance and other differences in parent emotional expression and positive affect, as well as parent and clinician social chitchat. Although most of these differences by race were due to differences in parent rather than clinician statements, they nonetheless suggest racial differences in the interpersonal care experienced by parents. Collectively, these associations are consistent with a pattern of social distance between predominantly White clinicians and Black compared with White parents, which may contribute to disparities in care quality and outcomes.

Verbal dominance was lower in visits with White compared with Black parents. Adjusting for parent demographics did not reduce this difference. Differences in verbal dominance ratios appeared to be primarily due to Black parents making fewer total statements in the unadjusted model. Higher verbal dominance during visits with patients of marginalized racial and ethnic backgrounds has been previously observed in other settings, including primary care and oncology,^7,8,9,10,11,12^ although the finding is not consistent across studies and settings.^13^ In primary care, verbal dominance and the similar metric of clinician-patient talk-time ratio have been previously associated with clinician scores on the Race Implicit Association Test,^14^ a measure of implicit racial bias,^4,15^ along with patient perceived past discrimination^15^ and decreased adherence to recommendations.^15^ Future research should further explore the role of implicit bias and the association of parent participation with decisions, care experiences, and clinical outcomes during and after presurgical consultations.

Parent emotional expression was more frequent in visits with White compared with Black parents in the unadjusted model. This may reflect differences in parent spontaneous expression of emotion, clinician effectiveness at eliciting parent emotions, or combined parent and clinician factors. In a study that analyzed communication between predominantly White clinicians interacting with patients with HIV,^16^ Black patients were less likely to spontaneously discuss their emotions than White patients, but clinicians were also more likely to block emotional expression and less likely to respond in ways that provided space for further discussion of the emotional issue. Inadequate emotional exchange is concerning in initial surgical consultations because clinicians may miss opportunities to respond to parent concerns that are relevant to treatment decision-making. Future research to elucidate the implications of unequal emotional expression for parent engagement in surgical decision-making includes exploring the content of emotional exchange using methods such as conversation analysis,^17^ Verona coding,^18^ or qualitative approaches and examining how emotional statements are associated with parent-reported and clinical outcomes.

Parent and clinician chitchat were less frequent in visits with Black compared with White parents. Black parents also had lower observer-reported positive affect than White parents, potentially signaling lower engagement or satisfaction. These findings suggest differences in rapport-building and the quality of the patient-clinician relationship. While there is limited research on nonmedical social talk, we reason that it strengthens relationships by conveying warmth and appreciation for the conversation partner, encouraging engagement and disclosure. Chitchat can potentially help clinicians see patients as unique individuals rather than members of groups, which may mitigate implicit bias and stereotyping.^19,20^

Education may have direct and indirect associations with communication. Education can contribute to higher health literacy and empowerment, resulting in more active participation. More education may also be associated with clinician communication implicitly by signaling less social distance between parents and clinicians. White parents reported higher educational attainment than Black parents in our study. However, racial differences in verbal dominance remained in regression models that adjusted for parent education, suggesting that individual differences in education-linked factors, such as patient understanding and empowerment, were not the sole factors associated with this dynamic. By contrast, adjusting for parent education attenuated the strength of association between parent race and the outcomes of chitchat and parent positive affect. These findings collectively suggest that structural forces, such as racial disparities in educational access, interact with interpersonal dynamics to produce communication differences in this setting. Thus, disparities in parent characteristics that are linked to education, such as knowledge, empowerment, and health literacy, should be taken into account when developing mitigation strategies.

Avenues for future research include examining the associations of visit communication with parent-reported outcomes and clinical outcomes, exploring how clinician bias may interact with the association between patient race and communication, correlating quantitative findings with qualitative analysis of communication, and further examining how communication with multiple clinicians during consultations is associated with the quality and equity of care. These results also suggest intervention approaches to promote equitable communication in this setting. Clinician behaviors that may help encourage parent participation include eliciting parent perspectives, asking open-ended questions, responding to parent emotions, and engaging in rapport-building social talk. Patient activation interventions that enhance parent empowerment and knowledge may also be valuable.

### Limitations

This study has several limitations. The study population included participants from a limited number of outpatient clinics from a single health system in the Baltimore, Maryland, region. Moreover, while multiple indications are represented, tonsillectomy and adenoidectomy accounted for 86.9% of analyzed visits. Our findings may therefore not be generalizable to all contexts or indications. This analysis assessed communication only with Black and White parents due to the relatively low representation of parents of other races or ethnicities in this study. Due to the small number of clinicians and their limited racial and ethnic diversity, we were unable to examine associations with clinician race and ethnicity or racial and ethnic concordance between clinicians and patients. We also had limited information about supporting clinicians, making it difficult to assess how their characteristics were associated with visit communication, although their communication was included in the analysis. Additionally, as an analytic method, RIAS captures general categories of medical task–focused and socioemotional communication but is more limited in measuring contextual and linguistic aspects of clinical communication.^5^

## Conclusions

In this cohort study of parent communication during pediatric surgical consultations, visits with Black parents had higher verbal dominance, less social chitchat, fewer parent emotional statements, and lower positive parent affect compared with visits with White parents. Verbal dominance was higher in visits with Black parents regardless of adjustment for parent sociodemographic factors. These findings signal opportunities for clinicians to encourage parent participation in decision-making for elective pediatric surgical procedures.

## Notes

Supplement 1. **eTable.** Unadjusted and Adjusted Associations of Parent Race and Communication With Primary Clinicians Only

Supplement 2.
Data Sharing Statement
