# Supplementary material for: Parent Race and Communication During Elective Pediatric Surgery Consultations
Source: JAMA Netw Open. 2025 Nov 11;8(11):e2542758. doi: 10.1001/jamanetworkopen.2025.42758 (PMC12606373; doi:10.1001/jamanetworkopen.2025.42758)
Supplement: Supplement 1. — eTable. Unadjusted and Adjusted Associations of Parent Race and Communication With Primary Clinicians Only [file jamanetwopen-e2542758-s001.pdf]

Supplemental Online Content

Lowe C, Beach MC, Saha S, Links AR, Boss EF. Parent race and communication during elective pediatric surgery consultations. *JAMA Netw Open*. 2025;8(11):e2542758. doi:10.1001/jamanetworkopen.2025.42758

eTable. Unadjusted and Adjusted Associations of Parent Race and Communication With Primary Clinicians Only

This supplemental material has been provided by the authors to give readers additional information about their work.

© 2025 Lowe C et al. *JAMA Network Open*.

Online-Only Table

eTable. Unadjusted and Adjusted Associations of Parent Race and Communication With Primary Clinicians Only

	Outcome	**Mean (SD) for Black Parents (n=57)[1]**	**Mean (SD) for White Parents (n=85)**	**Model 1. Unadjusted Race Effect (White = ref)**	**Model 2. Race Effect Adjusted for Demographics (Excl. Education)**	**Model 3. Race Effect Adjusted for Demographics (Incl. Education)**
		**Mean (SD)**	**Mean (SD)**	**Coefficient (95% CI)**	**Coefficient (95% CI)**	**Coefficient (95% CI)**
Overall	Verbal dominance	1.81 (0.75)	1.39 (0.45)	0.4 (0.08 to 0.71)	0.41 (0.1 to 0.73)	0.36 (0.06 to 0.66)
	Patient centeredness	0.37 (0.14)	0.37 (0.12)	0 (-0.04 to 0.03)	0.01 (-0.03 to 0.05)	0.02 (-0.01 to 0.06)
Clinician Behavior	Total clinician statements	184.32 (80.05)	154.87 (68.86)	14.46 (-18.42 to 47.34)	14.17 (-18.28 to 46.63)	15.43 (-15.66 to 46.53)
	Clinician facilitation/ activation statements	28.09 (14.27)	26.89 (14.25)	-0.35 (-4.04 to 3.34)	-0.5 (-5.69 to 4.68)	-0.81 (-5.16 to 3.54)
	Clinician emotional statements	9.91 (9.39)	7.09 (5.71)	1.67 (-1.55 to 4.88)	1.96 (-0.86 to 4.79)	2.27 (-0.95 to 5.49)
	Clinician chit-chat statements	3.04 (2.58)	3.84 (3.69)	-0.69 (-1.68 to 0.3)	-0.4 (-1.18 to 0.39)	-0.32 (-1.18 to 0.55)
Parent Behavior	Total parent statements	113.35 (56.22)	118.76 (53.3)	-4.79 (-22.41 to 12.83)	-10.24 (-24.38 to 3.89)	-2.48 (-22.76 to 17.81)
	Parent emotional statements	5.11 (5.49)	6.16 (4.5)	-0.78 (-1.88 to 0.32)	-1.04 (-2.65 to 0.58)	-2.53 (-5.99 to 0.93)
	Parent chit-chat statements	2.32 (3.05)	2.87 (2.88)	-0.56 (-1.39 to 0.28)	-0.27 (-0.94 to 0.4)	0.02 (-0.87 to 0.9)

© 2025 Lowe C et al. *JAMA Network Open*.
