# Supplementary material for: Parent Race and Communication During Elective Pediatric Surgery Consultations
Source: JAMA Netw Open. 2025 Nov 11;8(11):e2542758. doi: 10.1001/jamanetworkopen.2025.42758 (PMC12606373; doi:10.1001/jamanetworkopen.2025.42758)
Supplement: Supplement 2. — Data Sharing Statement [file jamanetwopen-e2542758-s002.pdf]

Data Sharing Statement

Lowe. Parent Race and Communication During Elective Pediatric Surgery Consultations. *JAMA Netw Open*. Published online November 11, 2025. doi:10.1001/jamanetworkopen.2025.42758

Data
Data available: No
